# Individual and community level factors associated with sexually transmitted infections among men in Tanzania: insights from the Tanzania demographic and health survey of 2022

**DOI:** 10.1186/s12879-024-09470-2

**Published:** 2024-06-12

**Authors:** Gosa Mankelkl, Seid Mohammed Abdu, Ebrahim Msaye Asefa, Altaseb Beyene Kassaw, Gashawu Abebe, Mekonnen Belete, Amare Abera Tareke, Alemu Gedefie, Beletu Kinfe

**Affiliations:** 1 Department of Biomedical Sciences, College of Medicine and Health Science, Wollo University, Dessie, Ethiopia; 2 Department of Medical Laboratory Sciences, College of Medicine and Health Sciences, Wollo University, Dessie, Ethiopia; 3 Department of occupational Health and safety, College of Medicine and Health Science, Wollo University, Dessie, Ethiopia

**Keywords:** Sexually transmitted infections, Multilevel, Tanzania

## Abstract

**Background:**

Sexually transmitted infections continue to be a significant public health issue on a global scale. Due to their effects on reproductive and child health as well as their role in facilitating the spread of HIV infection, sexually transmitted infections impose a heavy burden of morbidity and mortality in many developing countries. In addition, stigma, infertility, cancer, and an increased risk of HIV are the primary impacts of STIs on sexual and reproductive health. While numerous studies have been conducted in Tanzania to address this specific topic in various settings, the majority of them weren’t representative. Therefore, the main objective of this study was to use data from the most recent Tanzania Demographic and Health Survey in order to evaluate the individual and community-level factors associated with sexually transmitted infections among Tanzanian men at the national level.

**Methods:**

The most recent datasets from the Tanzania demographic and health survey were used for secondary data analysis. A total of 5763 men participated in this study. The recent Tanzania demographic and health survey provides data for multilevel mixed effect analysis on the variables that contribute to sexually transmitted infections among men in Tanzania. Finally, the percentage and odd ratio were provided, together with their 95% confidence intervals.

**Result:**

This study includes a total weighted sample of 5763 men from the Tanzania demographic and health survey. Of the total study participants, 7.5% of men had sexually transmitted infections in the last twelve months. Being married [AOR: 0.531, 95% CI (0.9014, 3.429)] was a factor that reduced the risk of sexually transmitted infections among men. On the other hand, being between the age range of 20 and 24 years [AOR: 6.310, 95% CI (3.514, 11.329)] and having more than one union [AOR: 1.861, 95% CI (1.406, 2.463)] were the factors that increased the risk of sexually transmitted infections among men.

**Conclusions:**

Men’s sexually transmitted infections have been associated with individual-level factors. So, the Tanzanian governments and the concerned stakeholders should provide special attention for men whose age range is 20–24 years old. Promoting marriages and limiting the number of sexual partners should be the main strategies to lower the risk of sexually transmitted infections among men in Tanzania.

## Introduction

Sexually transmitted infections (STIs) are a group of infections that are predominantly transmitted through unprotected sexual contact (vaginal, anal, and oral sex) with an infected person [1] and Some STIs can also be transmitted through skin-to-skin contact, from mother-to-child during pregnancy, childbirth, and breastfeeding [2]. Due to the impact that sexually transmitted infections have on reproductive and child health, as well as their role in promoting HIV infection, sexually transmitted infections impose a heavy burden of morbidity and mortality in many developing nations [3]. Globally, sexually transmitted infections other than HIV remain a major public health problem. Despite the strong association between STIs and HIV acquisition, STIs other than HIV have been overshadowed in recent years by the heightened public-health focus on HIV treatment. Naturally, STIs affect individuals who are part of partnerships and larger sexual networks, and in turn, the general population [4, 5].

In 2020, there were an estimated 374 million new infections of four curable sexually transmitted infections among people aged 15–49 years worldwide; about 26% (96 million) of these occurred in Africa [6]. Globally, common types of STIs in men include chlamydia, gonorrhea, trichomoniasis, and genital herpes [7]. Some of the most common STIs in men may not produce signs or symptoms [8]. These infections can have a serious impact on health status including pelvic inflammatory disease, stigmatization, infertility, cancers, increase the risk of HIV, adverse mental health, congenital deformities [6, 9, 10], and drug resistance is a major threat to reducing the burden of STIs worldwide [11]. There were an estimated 376 million new curable STIs annually. Additionally, more than 500 million people have genital infections with the herpes simplex virus, which predominantly occur in developing countries [10].

The government of the United Republic of Tanzania has endorsed several global commitments and their respective plans of action to improve quality of life and achieve the elimination of new HIV infections [12]. Additionally, in Tanzania, the national policy calls for recommending HIV testing to every person who comes to a health facility, regardless of their malady [13]. But little attention was given to other STIs due to the heightened public-health focus on HIV prevention and treatment in recent years compared to STIs. Although there are several studies that were done in Tanzania to address this particular topic in different settings, study periods, and sample sizes [14–17]. But most of them lack representativeness. So, the major objective of this study was to evaluate individual and community-level factors associated with sexually transmitted infections among men in Tanzania at the national level by using the recent Tanzania demographic and health survey. Since policymakers prefer country-level data and findings for designing and executing appropriate intervention programs at different levels to reduce sexually transmitted infections, the findings of this study would provide better evidence for policymakers and other stakeholders.

## Methods and material

### Study setting and period

Tanzania is an East African country situated just south of the Equator. The Tanzania mainland is bounded by Uganda, Lake Victoria, and Kenya to the north; by the Indian Ocean to the east; by Mozambique, Lake Nyasa, Malawi, and Zambia to the south and southwest; and by Lake Tanganyika, Burundi, and Rwanda to the west [18]. The Tanzania Demographic and Health Survey was implemented by the Tanzania National Bureau of Statistics (NBS) and the Office of Chief Government Statistician (OCGS) in collaboration with the Ministries of Health of Tanzania Mainland and Zanzibar and other stakeholders. The 2022 TDHS is the 7th Demographic and Health Survey conducted in Tanzania since 1991–92. Data collection took place from February to July 2022 in Tanzania, the Mainland and Zanzibar [19].

### Data source / data extraction

After permission was secured through an online request by explaining the aim of the study, the data for this analysis was obtained from the 2022 Tanzania Demographic and Health Survey, which can be accessed at the DHS portal (https://dhsprogram.com/data/dataset_admin/index.cfm.

### Study design and sampling procedures

**Fig. 1:**
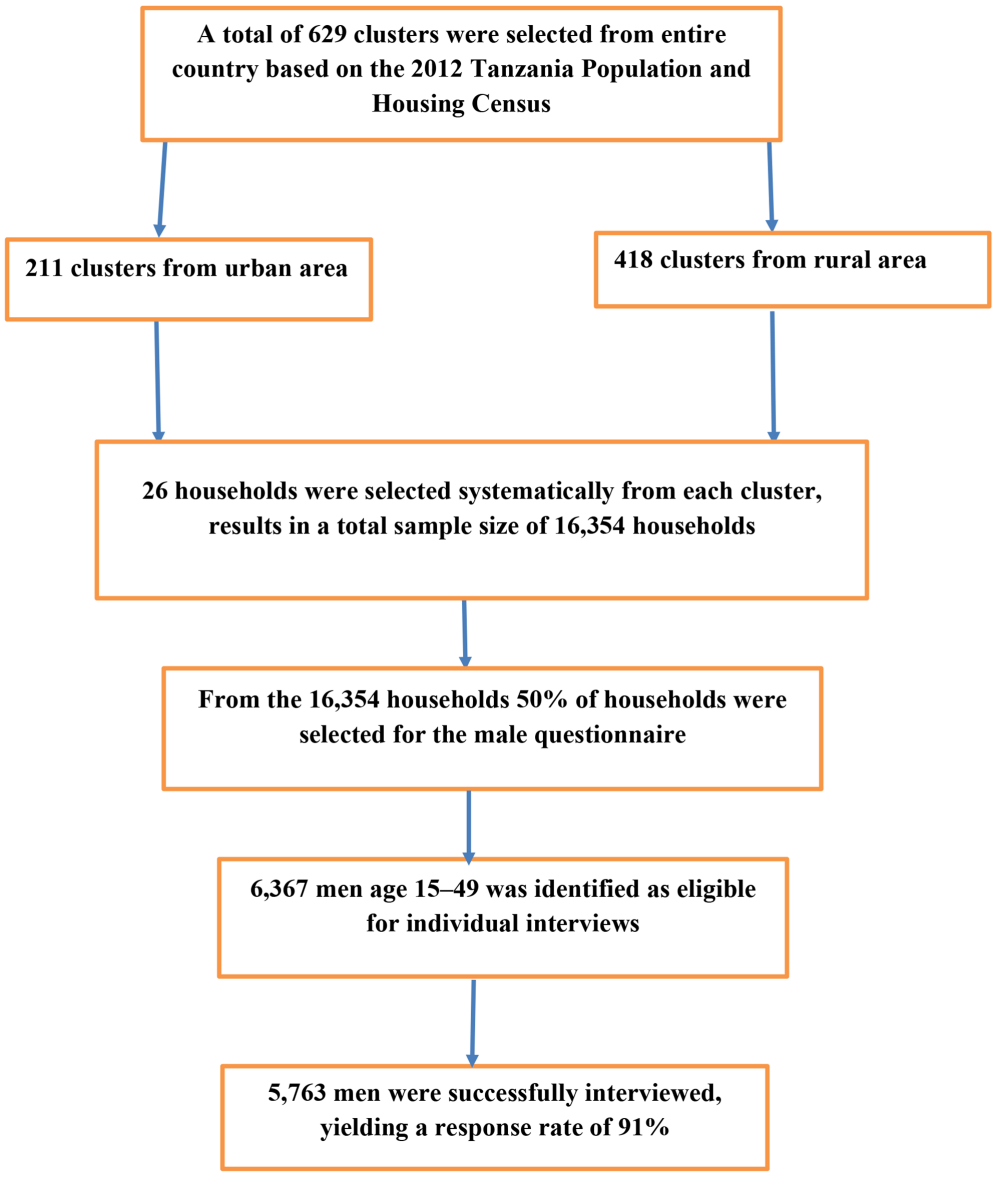
Showed that the diagrammatically presentation of the sampling procedures of men participants in Tanzania demographic survey 2022 [19]

A community-based cross-sectional study design was employed. The sample design for the 2022 TDHS was carried out in two stages and was intended to provide estimates for the entire country, for urban and rural areas in Tanzania Mainland, and for Zanzibar. The first stage involved the selection of sampling points (clusters) consisting of enumeration areas (EAs) delineated for the 2012 Tanzania Population and Housing Census (2012 PHC). The EAs were selected with a probability proportional to their size within each sampling stratum. A total of 629 clusters were selected. Among the 629 EAs, 211 were from urban areas and 418 were from rural areas. In the second stage, 26 households were selected systematically from each cluster, for a total anticipated sample size of 16,354 households for the 2022 TDHS. In a subsample of half of all households selected for the survey, all men ages 15–49 were eligible to be interviewed if they were either usual residents or visitors in the household on the night before the survey interview. In the subsample (50% of households) of households selected for the male questionnaire, 6,367 men ages 15–49 were identified as eligible for individual interviews, and 5,763 were successfully interviewed, yielding a response rate of 91% [19]. The final variable for this study was sexually transmitted infection among men. This study employed a total of 5,763 samples(Fig. 1).

### Study variables

#### The outcome variable

for this study was sexually transmitted infection. If the men hadn’t had any sexually transmitted infections in the last 12 months, which were labeled “no” and coded as “0,” that means the respondent hadn’t had STIs in the last 12 months, and if the men had sexually transmitted infections in the last 12 months, which were labeled “yes” and coded as “1,” that means the respondent had a history of STIs in the last 12 months.

#### Individual level variables

Age groups, educational status, marital status, sex of household head, wealth index, respondent circumcised, number of unions, reading newspapers, listening to radio, watching television, using the internet, using health insurance, currently working, and information about STI.

#### Community level variables

place of residence.

### Data management and analysis

To accommodate the intricate survey design, we took weighting, stratification, and clustering into consideration throughout the entire analysis process. To arrive at this result, the standard weights of the men and the total number of men in the country were divided by the corresponding survey sampling proportion. Data extraction, recoding, descriptive analysis, and analytical analysis were performed using STATA version 14.

Due to the hierarchical structure of the demographic and health survey data, a multilevel mixed effect model analysis was employed. The variation was assessed using the Interclass Correlation Coefficient (ICC), and Akaike’s information criterion (AIC) was used to evaluate model fitness and model comparison. Four models were built for this multistage investigation. The first model was built without independent factors; it is possible to determine the extent of cluster variation in the STI among men. The second model was fitted with individual-level factors alone. Community-level characteristics were incorporated into the third model. Finally, the fourth model took into account factors at both the individual and community levels. The model with the lowest deviance (AIC) value provided the best-fitting model. Then, bivariate analysis was employed in order to select the factors for multivariate analysis. In the multivariate analysis, variables with *p* < 0.05 significance levels were considered to be significant predictors of STI among men. Finally, the percentage and odd ratio were provided, together with their 95% confidence intervals.

### Ethical consideration

For this particular investigation, an ethical review and participant agreement were not required because the demographic and health survey program utilized secondary, easily accessible survey data. We requested permission from the DHS Program to use the data we obtained from their website, and they gave it to us.

## Results

### Socio-demographic characteristics of the participants

In all, 5763 men participated in this investigation. There were about 1444 people (25.1%) in the 15–19 age range. 3825 (66.4%) of the respondents were rural residents; 3134 (54.4%) attended primary school; 2621 (45.5%) were married; 4807 (83.4%) were households headed by men; and 3110 (54.0%) were poor in wealth; 2314 (40.2%) read magazines; 4570 (79.3%) listened to the radio; 4437 (77.0%) watched television; 1416 (24.6%) used the internet; 389 (6.8%) had health insurance, and 4951 (85.9%) had undergone circumcision. 699 (12.1%) had multiple unions; 1517 (26.3%) had recently engaged in sexual activity; and 4689 (81.4%) were employed. 4555 (79.05%) had information about STIs, and 430 (7.5%) had experienced a STI in the last 12 months (Table 1).

**Table 1:** Socio demographic characteristics of interviewed men in Tanzania 2022

Characteristics (*n* = 5763)	Categories	Frequency	Percentage
Age groups	15–19	1444	25.1
	20–24	934	16.2
	25–29	850	14.8
	30–34	765	13.3
	35–39	694	12.0
	40–44	608	10.5
	45–49	469	8.1
Place of residence	Urban	1938	33.6
	Rural	3825	66.4
Educational status	No education	574	10.0
	Primary	3134	54.4
	Secondary	1858	32.2
	Higher	197	3.4
Marital status	Never in union	2518	43.7
	Married	2621	45.5
	Others	625	10.8
Sex of house hold head	Male	4807	83.4
	Female	955	16.6
Wealth index	Poor	3110	54.0
	Middle	1355	23.5
	Rich	1297	22.5
Reading newspaper	No	3449	59.8
	Yes	2314	40.2
Listening radio	No	1193	20.7
	Yes	4570	79.3
Watching television	No	1326	23.0
	Yes	4437	77.0
Using internets	No	4347	75.4
	Yes	1416	24.6
User of health insurance	No	5374	93.2
	Yes	389	6.8
Respondent circumcised	No	812	14.1
	Yes	4951	85.9
Number of unions	Once	2368	41.1
	More than once	699	12.1
	never in union	2696	46.8
Recent sexual activity	Never had sex in last 4 weeks	4246	73.7
	Active in last 4 weeks	1517	26.3
Currently working	No	1074	18.6
	Yes	4689	81.4
Had information about STI	No	1208	21.0
	Yes	4555	79.0
Had any STI	No	5333	92.5
	Yes	430	7.5

### Model comparison

The intraclass correlation coefficient (ICC) in the null model showed that among men, there was a variation in sexually transmitted infections of 14.02% accounted for by inter-cluster differences. The variance in sexually transmitted infections among men is described by variables at the individual level in 13.97% of differences. The difference in sexually transmitted infections among men is accounted for by community-level variables at 9.83%. In the end, 7.87% of the variations among men were caused by variables at the individual and community levels. As a result, it was determined that Model IV, which included factors at both the individual and community levels, had the lowest deviance (AIC) value. Therefore, Model IV is the best-fitted model, and every interpretation and report were made based on this model (Table 2) .

**Table 2:** revealed the random effect of sexually transmitted infection

Parameters	model I	model II	model III	Model IV
ICC (%)	14.02	13.97	9.83	7.87
Model fitness				
**AIC**	1062.19	1055.83	996.56	870.23

### Factors analysis associated with sexually transmitted infections

The results of the bivariable analysis using model IV showed that among men, sexually transmitted infections were statistically and significantly associated with age groups, marital status, wealth index, reading newspapers, listening to radio, watching television, using the internet, number of unions, and currently working. The results of the multivariate analysis demonstrated that sexually transmitted infections were statistically and significantly associated with age groups, marital status, listening to radio, and the number of unions.

The finding from this study shows that the odds of sexually transmitted infections between the age range of 20 and 24 years old were 6.310 times more likely [AOR: 6.310, 95% CI (3.514, 11.329)] compared to men whose ages were between 15 and 19-years old’s. The odds of sexually transmitted infections among men who were married were 0.531 times less likely [AOR: 0.531, 95% CI (0.9014,3.429)] relative to men who were not married. The odds of sexually transmitted infections among men who were listening to radio were 1.400 times more likely [AOR: 1.400, 95% CI (1.012, 1.937)] compared to their counterparts. The odds of sexually transmitted infections among men who had more than one union were 1.861 times more likely [AOR: 1.861, 95% CI (1.406,2.463)] compared to men who had only one union (Table 3).

**Table 3:** Factors associated with sexually transmitted infections among men in Tanzania 2022

Variable (*n* = 5763)	Categories	STI		COR with 95% CI; *P*-value	AOR with 95% CI; *P*-value
		No	Yes		
Age groups	15–19	1,439	18	1	1
	20–24	903	56	4.957(2.896,8.486) ***	4.161(2.382,7.268) ***
	25–29	761	85	8.929(5.329,14.960) ***	6.310(3.514,11.329) ***
	30–34	662	60	7.245(4.245,12.368) ***	5.061 (2.706,9.466) ***
	35–39	627	59	7.522(4.401,12.857) ***	5.071(2.682,9.588) ***
	40–44	573	48	6.696(3.862,11.610) ***	4.340(2.254,8.357) ***
	45–49	431	41	7.604(4.324,13.374) ***	4.994(2.562,9.733) ***
Place of residence	Urban	1,749	134	1	
	Rural	3,647	233	0.834(0.669,1.039)	
Educational status	No education	560	37	1	
	Primary	2,756	222	1.219(0850,1.746)	
	Secondary	1,913	100	0.791(0.536,1.167)	
	Higher	167	8	0.725(0.331,1.587)	
Marital status	Never in union	2,483	90	0.234(0.172,0.319) ***	0 0.501(0.263,0.954) **
	Married	2,349	190	0.524(0.400,0.686) ***	0.531(0.9014,3.429) ***
	Others	564	87	1	1
Sex of house hold head	Male	4,493	319	1.335(0.977,1.824)	
	Female	903	48	1	
Wealth index	Poor	2,931	185	1	1
	Middle	1,236	105	1.345(1.049,1.725) **	1.159(0.888,1.513)
	Rich	1,229	77	0.992(0.754,1.305)	0.797(0 0.574,1.106)
Reading newspaper	No	3,496	211	1	1
	Yes	1,900	156	1.360(1.097,1.685) ***	1.136(0.896,1.439)
Listening radio	No	1,272	52	1	1
	Yes	4,124	315	1.868(1.384, 2.522) ***	1.400(1.012,1.937) *
Watching television	No	1,319	63	1	1
	Yes	4,077	304	1.561(1.181,2.062) ***	1.339(0.985,1.821)
Using internets	No	4,184	262	1	1
	Yes	1,212	105	1.383(1.093, 1.750) ***	1.175(0.883,1.563)
User of health insurance	No	5,066	352	1	
	Yes	330	15	0654(0.385,1.109)	
Respondent circumcised	No	801	60	1	
	Yes	4,595	307	0.891(0.669,1.188)	
Number of unions	Once	2,124	170	1	1
	More than once	598	91	1.901(1.312,2.491) ***	1.861(1.406,2.463) ***
	never in union	2,674	106	0.495(0.386,0.635) ***	1.134(0.678,1.950)
Recent sexual activity	Never had sex in last 4 weeks	4,074	271	1	
	Active in last 4 weeks	1,322	96	1.091(0. 857,1.389)	
Currently working	No	1,105	30	1	1
	Yes	4,291	337	1.892(1.979,4.226) ***	1.281(0.847, 1.937)

COR = crudes odds ratio, AOR = adjusted odds ratio; CI-confidence interval

Statistically significant at *P < 0.05; **P < 0.01; ***P < 0.001

## Discussions

The prevalence’s of sexually transmitted infections among men in united republic of Tanzania were 7.5%. This finding was in line with study which were conducted in Ghana(6%) [20], Kenya 7.4% [21], in Africa (7.7%) [22]. This finding was lower than the study which conducted in Liberia (13.4%) [23]. This finding was higher than the study which was conducted in Guinea (5.9%) [24]. The first possible reasons for this variation were a difference in the study period, method of estimation, sample size, socioeconomic status, and the geographic location of the study area, which could be the cause of the discrepancy between our findings and those of the previously mentioned studies. The second possible reason for this variation were socio-cultural practices and beliefs that affected health-seeking behaviors towards STIs. Additionally, STI patients still resort to the use of traditional methods in treating their infections due to their traditional ethno-medical beliefs.

The finding from this study shows that the odd of sexually transmitted infections between the age range of 20–24 years old were more likely compared to men whose ages were between 15 and 19 years old’s. This finding was supported by the study which was conducted in Ghana [20] and Ethiopia [25]. Adolescents and young adults are particularly vulnerable to sexually transmitted infections [26]. But in cases of our finding, adolescents, particularly those between the ages of 20 and 24, were more likely compared to men whose ages were between 15 and 19 years old. This might be in the age groups of 20–24 years; most of the young men have become independent or started living independently compared to the young men between the ages of 15 and 19 years old. This makes them more susceptible to having multiple sexual partners, peer pressure, difficulty accessing health services, feeling uncomfortable discussing their sexual lives with a doctor, and lacking insurance, which can make it more challenging for young men (20–24 years old) to get sexually transmitted infection testing and treatments compared to young men between the ages of 15 and 19 years old. On the other hand, most of the time, young men who were found between the ages of 15 and 19 were living with their parents. As a result, they became less prone to risky sexual behaviors, peer pressure, and their health insurance was covered by their parents.

The odd of sexually transmitted infections among men who were married were less likely relative to men who were others (living with partner, widowed, divorced, and no longer living together). This finding was supported by the study which was conducted in Ethiopia [27, 28], and in united states [29]. A potential explanation is that societal norms and expectations related to marriage, which value fidelity and monogamy, are frequently linked to marriage. These social norms have the potential to discourage men from engaging in risky behavior by discouraging extramarital affairs. This could be more explained by the fact that in Tanzania, religious people play spiritual and advisory roles in the sexual activities of adolescents. The standing norm drawn from religious and social beliefs is “no sex before marriage [30]. Those norms discourage men from engaging in risky sexual behaviors before marriage, which results in a low incidence of STIs among married men. Moreover, marriage can foster emotional closeness and fulfillment, which lessens the perception of the need to pursue satisfaction through risky extramarital sex [31, 32]. Additionally, being married predisposes one to take preventive measures against the spread of sexually transmitted infections. In contrast to this, never-married youths are repeatedly exposed to unprotected sex at a young age, which increases the risk of contracting STIs [33].

The odd of sexually transmitted infections among men who were listening to radio were more likely compared to their counterparts. This could be explained from the perspective that often these media platforms show health promotional messages that encourage people to go for testing, and by so doing, many men become aware of their STI status [34–36]. So. The reporting and testing rate of STIs became high among men who were exposed to those media. Another reason for this finding could be that some media carry sexually explicit content that could entice adolescent men to engage in risky sexual behaviors, which increases their chances of reporting STIs [23, 37, 38]. This finding was contradicted by the studies which were conducted in Ethiopia [39], and in Ghana [20]. The possible reason might be the media is broadcasting STI prevention information. The information may have enabled men to avoid unsafe sexual practices to protect themselves from STIs and HIV/AIDS [40, 41].

Men who were in multiple relationships had a higher probability of contracting sexually transmitted infections than men who were in just one relationship. This finding was supported by the studies which were conducted in Kenya [39, 42]in south Africa [26], in Ethiopia [43] and Britain [44]. It is well established that having multiple sexual partners increases the risk of acquiring STIs. Because of this, the two recommended methods to prevent STIs are abstinence and becoming faithful to one sexual partner [45, 46].

### Strengths and limitations of this study

The study had many strengths, for instance; the DHS has a similar design with identical variables in a different environment; the result may, therefore, be applicable to other similar locations. The study used a sufficiently large sample size at the national level to ensure its representativeness. Yet, we would like to assure our reader that a few limitations need to be taken into account. Recall bias is one of the potential drawbacks, especially for retrospective data based on past experiences. Additionally, the magnitude of the bias is often unknown, and correcting for the bias is difficult. Furthermore, this study was a cross-sectional study. It doesn’t show temporal relationships between independent and dependent variables, which may affect the deterrent factors of sexually transmitted infections among men. The final potential limitation of this study was the use of a community-based design that depends on their history of STI in the last 12 months and didn’t implement the laboratory diagnosis on the spot of data collection.

### Conclusion and recommendation’s

Men’s sexually transmitted infections have been associated with individual-level factors. So, the Tanzanian governments and the concerned stakeholders should provide special attention for men whose age range is 20–24 years old. Promoting marriages and limiting the number of sexual partners should be the main strategies to lower the risk of sexually transmitted infections among men in Tanzania.

## Data Availability

The data were obtained from the Tanzania Demographic and Health Survey 2022, which was found at the DHS portal (https://dhsprogram.com/data/dataset_admin/index.cfm).
